# Facilitators and barriers to implementing electronic patient-reported outcome and experience measures in a health care setting: a systematic review

**DOI:** 10.1186/s41687-023-00554-2

**Published:** 2023-02-14

**Authors:** Ben G. Glenwright, Joshua Simmich, Michelle Cottrell, Shaun P. O’Leary, Clair Sullivan, Jason D. Pole, Trevor Russell

**Affiliations:** 1 Physiotherapy Department, Cairns Hospital, Cairns Hinterland and Hospital Health Service, Orthopaedic Ward, D6, Cairns Hospital, 165 The Esplanade, Cairns, QLD 4870 Australia; 2 School of Health and Rehabilitation Sciences, University of Queensland, Brisbane, Australia; 3 RECOVER Injury Research Centre, University of Queensland, Brisbane, Australia; 4 Centre for Health Services Research, University of Queensland, Brisbane, Australia; 5 Physiotherapy Department, Royal Brisbane and Women’s Hospital, Brisbane, Australia

## Abstract

**Objective:**

This systematic literature review aimed to identify factors that influence the implementation of electronic patient-reported outcome measures (ePROMs) and patient-reported experience measures (ePREMs) in healthcare settings.

**Introduction:**

Improvements in health care through increased patient engagement have gained traction in recent years. Patient-reported outcome measures (PROMs) and patient-reported experience measures (PREMs) are tools used to improve the quality of care from the patient perspective. The influence of implementing PROMs and PREMs using electronic information systems (ePROMs and ePREMs) is not well understood.

**Inclusion criteria:**

Studies with information related to the implementation of ePROMs and/or ePREMs with a focus on health-related services, irrespective of provider type, were included.

**Methods:**

A literature search of peer-reviewed databases was conducted on the 24th of January 2022 for articles about barriers and facilitators of the implementation of ePROMs/ePREMs in healthcare settings. Two reviewers independently extracted relevant findings from the included studies and performed a descriptive code-based synthesis before collaboratively creating a final consensus set of code categories, which were then mapped to the consolidated framework of implementation research (CFIR). Study quality was appraised using a mixed-methods appraisal tool (MMAT).

**Results:**

24 studies were eligible for inclusion in the screening of 626 nonduplicate studies. Quality assessment using the MMAT revealed that 20/24 studies met at least 60% of the MMAT criteria. Ninety-six code categories were identified and mapped to the constructs across all CFIR domains.

**Conclusion:**

To guide the effective implementation of ePROMs/ePREMs in healthcare settings, factors shown to influence their implementation have been summarised as an implementation checklist for adoption and use by clinicians, organisations, and policymakers.

**Supplementary Information:**

The online version contains supplementary material available at 10.1186/s41687-023-00554-2.

## Background

Capturing patient outcomes and experiences is critical to enabling ongoing review and improvement of healthcare services [1]. Patient experiences can improve health care services towards greater patient-centred care by understanding the nuanced interactions with a health service and its practitioners. Patient outcomes can be used to capture a person’s perception of their own health, such as their quality of life, daily functioning, symptoms, and other aspects of their health and well-being. Evaluating both outcomes and experiences of a patient’s health journey permits a holistic insight into healthcare services and ensures they are valued with a patient-centred focus.

Healthcare digitisation has provided an unprecedented opportunity to collect patient information in more recent years via an electronic medium in the form of electronic patient-reported outcome measures (ePROMs) and electronic patient-reported experience measures (ePREMs). ePROMs are digital questionnaires measuring patients’ views on their health status, symptoms, daily functioning, quality of life, and other characteristics of health and well-being [2]. ePREMs, on the other hand, are digital questionnaires measuring patients’ perceptions of their experiences while receiving care [2]. A 2019 study suggested ePROMs, when compared to traditional paper-based patient-reported outcome measures (PROMs), improve data quality, result in similar or faster completion times, lower costs, and facilitate clinical decision-making and symptom management [3]. However, disadvantages of digital delivery include privacy concerns, large initial financial investment and the ‘digital divide’ disadvantaging patients who are not digitally engaged [3]. Furthermore, there is limited information on the use of ePREMs, and the facilitators and barriers to implementation [4].

ePROMs/ePREMs allow for patient monitoring across all levels of the healthcare landscape, from individual clinician-patient ‘point-of-care’ interactions (micro level) to population surveillance and informing policy (macro level) [5]. Integration of patient-reported data with clinical and service delivery data can enable a holistic view of patients’ overall care journey, while concurrently enabling the monitoring of service performance to identify gaps and opportunities for enhancement, creating a positive feedback loop for improvement [5, 6]. Despite the apparent opportunities provided by digital technology, in practice the implementation and uptake of ePROMs, and especially ePREMs, remains sparse and inconsistent. Barriers to ePROM uptake have been reported, such as insufficient stakeholder engagement and training, a lack of interoperability with existing clinical information systems, and data management [7–9]. There is little known about whether the barriers and facilitators for the implementation of ePREMs and ePROM overlap.

To address these knowledge-to-practice gaps in the utilisation of ePROMs and ePREMs across healthcare settings, this study aimed to systematically review the literature to a identify facilitators and barriers to implementing both ePROMs and ePREMs in a healthcare setting, and map the identified facilitators and barriers to the Consolidated Framework of Implementation Research (CFIR) determinant framework. CFIR is a commonly used implementation science framework to facilitate the design, evaluation, and implementation of evidence-based interventions by systematically assessing potential facilitators and barriers for implementing an intervention at all levels of the healthcare system, including individual, organisational, and beyond [10–13]. In comparison to other frameworks, such as the theoretical domains framework, CFIR was chosen for its comprehensive examination of the various tiers of healthcare and the various implementation strategies necessary for success. The study also aimed to summarise the findings into an actionable checklist that defines best implementation practices.

## Review question

What factors (facilitators and barriers) are currently identified in the literature as influencing the implementation of ePROMs and ePREMs in health care?

### Inclusion criteria

The following eligibility criteria were developed to frame the review, based on a modification of the criteria used in a previous systematic literature review of PROM implementation facilitators and barriers [14].

#### Population

Studies including patients, clinicians, commissioners, or managers of health-related services. Commissioners could be representatives of either local or national agencies that finance health-related services (e.g., policymakers).

#### Phenomena of interest

Studies that investigated factors reported to influence the implementation of ePROMs and/or ePREMs.

#### Context

Studies investigating health-related services, irrespective of patient populations, providers, sectors of health care or country.

Articles that met all the following inclusion criteria were included in the review:

1. Include studies reporting facilitators and barriers (or factors) that influenced the implementation of ePROMs and/or ePREMs.
2. Have a digital delivery method that involves the sharing of information including data and content among mobile phones, computers, and tablets.

Articles were excluded if they were:

1. Protocol papers (no results reported).
2. Studies on the issues of implementing ePROMs and/or ePREMs in research contexts (e.g., clinical trials) rather than in a clinical context.
3. Development or usability studies and other pre-implementation research studies.
4. Reports indicating fewer than 75% of participants (e.g., clinicians undertaking surveys) actively use ePROMs and/or ePREMs.

*Type of studies***:** This review considered quantitative, qualitative, and mixed-methods studies.

## Methods

This systematic review was registered in PROSPERO (registration number: CRD42022295392) and follows the Preferred Reporting Items for Systematic Reviews and Meta-Analyses (PRISMA) guidelines for reporting systematic reviews [15]

### Search strategy

The search strategy keywords included patient-reported outcome measures; patient-reported experience measures; implementation; and electronic, digital, and mHealth. The full search strategy is reported in Additional File 1.

### Information sources

#### Study selection

An electronic data search of five electronic databases (PubMed, CINAHL, PsycINFO, Web of Science, and Scopus) was conducted between the database inception and the search date (24th of January 2022). Search results were exported to Covidence [16], and duplicate entries removed. Manual screening of the reference lists of all included studies was performed to identify articles not identified in the database search. Two reviewers (BG and JS) independently screened the study titles and abstracts for inclusion in the full-text review, and then reviewed the full-text articles for final inclusion in the systematic review. Disagreements regarding article eligibility were resolved through discussion between the two reviewers. Where agreement could not be reached, a third reviewer (MC) adjudicated as necessary.

#### Assessment of methodological quality

Two reviewers (BG and JS) utilised the Mixed-Methods Appraisal Tool (MMAT) to assess the methodological quality of the included studies [17]. The MMAT contains a checklist of five sets of questions, each corresponding to a specific study design category. Each question must be answered as either “yes,” “no,” or “cannot tell.”. According to Coates et al. [18], studies were considered of high quality when meeting 100% of the criteria, moderate quality when meeting 80–99% of the criteria, average quality when meeting 60–79% of the criteria, low quality when meeting 40–59% of the criteria, and very low quality when meeting < 39% of the criteria. Both authors determined consensus quality ratings for each study, from which the overall percentage score for each study was derived. Agreement between the two reviewers for the quality assessment was calculated as both percent agreement and first-order agreement coefficient, as defined by Gwet’s AC1 [19, 20], and computed using the *irrCAC* (version 1.0) package [20], in R software (version 4.0.3) [21].

#### Data extraction

A single reviewer (JS or BG) extracted data pertaining to the characteristics of the included studies, such as the country of origin, clinical setting and type of study. Studies were categorised based solely on the relevant data extracted for this review (e.g., some studies collected both quantitative and qualitative data but if only the qualitative data was extracted these studies were placed in the qualitative category).

#### Data transformation

A descriptive code-based synthesis of the results was performed using qualitative descriptive coding to identify and synthesise the relevant findings of the included studies. Two reviewers (BG, JS) independently extracted findings from the included studies stating factors relevant to the implementation of ePROMs/ePREMs (e.g., specific participant quotes, themes derived by the authors of the included study, discussions points and ‘qualitized’ descriptions of findings from quantitative results).

#### Data synthesis and integration

The two reviewers coded all extracted data into bottom-level codes. Using content analysis [22], each reviewer independently grouped their set of bottom-level codes into top-level code categories using an inductive approach. Through discussion, the two reviewers collaboratively created a final consensus set of code categories and mapped these to relevant CFIR constructs.

Many of the resulting code categories were bidirectional, so they could be considered facilitators or barriers to implementing ePROMs/ePREMs in healthcare settings, depending on their execution. The two reviewers classified code categories as facilitators if more than half of the codes in that category were deemed to be facilitators (and, similarly, for barriers).

## Results

### Study inclusion

**Fig. 1:**
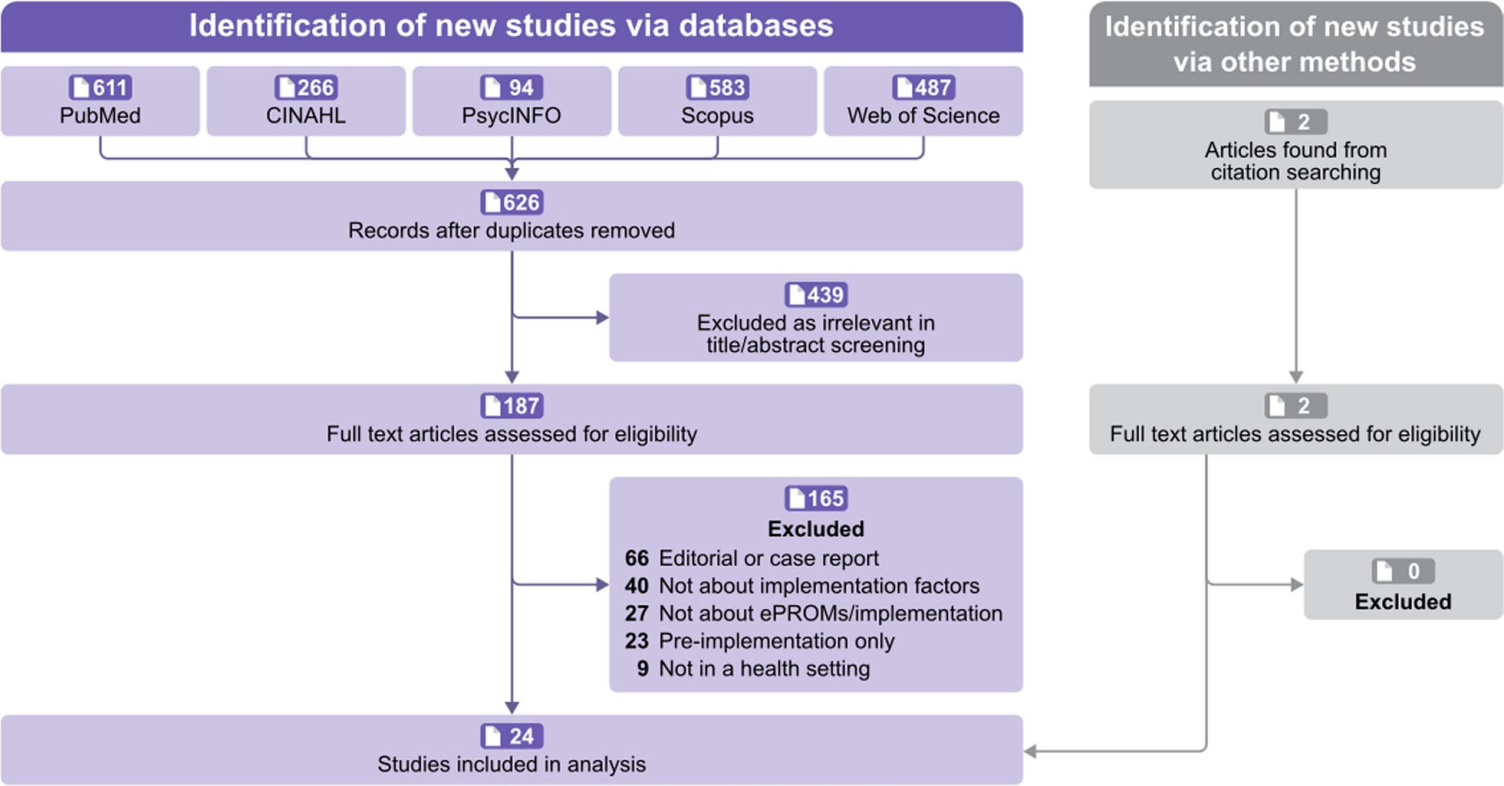
PRISMA flow diagram of study selections

Figure 1 shows the preferred reporting items for the systematic reviews and meta-analyses (PRISMA) [23]. The search strategy yielded 626 records, of which 187 full-text were assessed for eligibility and 24 studies met the inclusion criteria.

### Methodological quality

All articles were assessed and assigned to a category under the MMAT [17]. As shown in Table 1, eight studies were rated as high quality [24–30], seven as moderate quality [7, 8, 31–35], five as average quality [9, 36–39], two as low quality [40], and two as very low quality [4, 41]. No study was excluded based on quality assessment. The percent agreement for the quality assessment (excluding the MMAT screening questions) between the two reviewers was 81%, with a Gwet’s AC1 of 0.79 (95%CI 0.72–0.86) indicating good agreement. Full MMAT scores for all included studies, are reported in Additional File 2.

**Table 1:** Characteristics of included studies

References	Intervention	Study design	Quality rating (%)	Country	Clinical setting
*ePREMs only*					
DeRosis [4]	ePREM	Qualitative	20	Italy	General hospital
*ePROMs only*					
Fredericksen [24]	ePROM	Qualitative	100	USA	Community health
Nielsen [26]	ePROM	Qualitative	100	Denmark	Gastroenterology
Rotenstein [38]	ePROM	Qualitative	100	USA	Oncology
Taliercio [43]	ePROM	Qualitative	100	USA	Dermatology
Unsworth [29]	ePROM	Qualitative	100	UK	Counselling/psychotherapy
Zhang [30]	ePROM	Qualitative	100	USA	Orthopaedic and oncology
Spaulding [28]	ePROM	Qualitative	93	USA	Psychiatry
Kwan [7]	ePROM	Qualitative	80	USA	General clinical practice
Papuga [9]	ePROM	Qualitative	60	USA	Orthopaedics
Schepers [8]	ePROM	Qualitative	60	Netherlands	Paediatric oncology
Trautmann [39]	ePROM	Qualitative	60	Germany	Oncology
Short [27]	ePROM	Observational quantitative	73	Canada + USA	Community outpatients
Li [37]	ePROM	Observational quantitative	60	Canada	Oncology
Bärlund [42]	ePROM	Observational quantitative	40	Finland	Oncology
Hanmer [40]	ePROM	Observational quantitative	40	USA	Paediatrics
Teela [35]	ePROM	Mixed methods	100	Netherlands	General hospital
Burton [32]	ePROM	Mixed methods	93	USA	General hospital
Duman-Lubberding [34]	ePROM	Mixed methods	93	Netherlands	Oncology
Dronkers [33]	ePROM	Mixed methods	86	Netherlands	Oncology
Amini [31]	ePROM	Mixed methods	80	Netherlands	General clinical practice
Howell [36]	ePROM	Mixed methods	60	Canada	Oncology
Bhatt [41]	ePROM	Mixed methods	20	USA	Orthopaedics
*ePROM* + *ePREM*					
Krawczyk [25]	ePROM + ePREM	Qualitative	100	Canada	Palliative care

### Characteristics of the included studies

The full details of the study’s characteristics are presented in Table 1. All studies were published after 2012, and conducted in eight countries: Netherlands [8, 31, 33–35], Finland [42], Denmark [26], United Stated of America [7, 9, 24, 27, 28, 30, 32, 38, 40, 41, 43], Italy [4], Canada [25, 27, 36, 37], Germany [39], and the United Kingdom [29]. The majority of studies (22/24) investigated ePROMs in isolation, one study investigated only ePREMs [4], and one study investigated both ePROMs/ePREMs [25]. The most common clinical setting was oncology, with 29% (7/24) of the included studies [8, 33, 34, 36–39, 42], followed by general hospital settings (3/24, 12%) [4, 32, 35]. Study designs were primarily qualitative (13/24) or mixed methods (7/24), but also included four observational quantitative studies. Across both qualitative and mixed-methods study designs, qualitative data were collected through interviews in 12 studies [24–26, 28–30, 32–34, 38–40, 43], focus groups in six studies [7, 9, 25, 29, 32, 36], and open-ended survey questions in five studies [4, 8, 31, 35, 38]. In studies that included a quantitative component, quantitative data were collected via close-ended survey questions in 11 studies [8, 27, 31–38, 40], a workflow audit in only one study [40], and Q-sorting in only one study [32].

### Findings of the review: facilitators and barriers to implementing ePROMs/ePREMs

A total of 96 code categories were generated, of which 60 were classified as facilitators and 36 as barriers. Code categories were subsequently mapped to 26 constructs across all five CFIR domains. Table 2 provides quotes for the five most prevalent categories (facilitators or barriers) mapped to each CFIR domain with the prevalence of each code category defined by the number of studies containing at least one code for each individual category. A full list of all identified categories mapped to individual CFIR constructs can be found in Additional File 3: Table S1.

**Table 2:** Example quotes for the top five most prevalent categories mapped to each CFIR domain (including ties)

Facilitator (F)/Barrier (B)	Category prevalence (no. of studies)	Example quotes
*Domain 1: Intervention characteristics* Key attributes of interventions that influence the success of implementation		
ePROMs/ePREMs monitor changes of patients (F)	12	PROMs also allowed physicians to “track any symptom over time from visit to visit”. [30]
Graphical visualisations of ePROM results to see trends (F)	10	"Graphical overview over time: clear" […] "Graphical aspect is useful" […] "Nice trend of the wellbeing over time". [8]
ePROMs time burden (length, repetition, or timing) (B)	10	"Sometimes patients seem a bit overwhelmed by having to answer all of the questions and the broad scope of it. Some of these folks might be better off just skipping it". [24]
ePROMs/ePREMs facilitate extracting information that might be overlooked or not be uncovered in consultation (F)	10	"During follow-up setting very welcome especially for adolescent who then often struggle with return to society and quality of life problems emerge more often" … "Information that becomes accessible with children that have difficulties talking" […] "With select group of patients where contact does not run smoothly". [8]
Lack of reliable and robust software and hardware (B)	8	Technical aspects "It takes effort to log in" "I do not receive an automatic message when patients have completed PROMs" "I have to print the ePROfile, because we do not have computers in the consultation room". [35]
User-friendly software and technology (F)	8	I found the care monitor platform user-friendly. [31]
Real-time access to ePROMs/ePREMs completion status and results data prior to, and during, consult (F)	8	Clinicians have real-time access to the results, which are graphically displayed inside the hospital information system. [33]
*Domain 2: Outer setting* Includes the features of the external context or environment that might influence implementation		
ePROMs/ePREMs amplify patient's voice, facilitate patient-centred care and shared decision-making (F)	13	"For those who want another way to voice their experiences, it’s fantastic because a lot of people... you know, by the time they kind of come to us within their journey of health care and transitioning through the disease process, a lot of people don’t feel like they’ve been listened to". [25]
ePROMs allow patients to better communicate and prioritise in clinic visits (F)	12	Focuses the session. The use of the measures provided a focus for short-term work: “I found if when I asked a question about ‘which question stood out for you’, not regarding the score so much, then we can talk about it in that way and bring focus to that and that was helpful”. [29]
Completing ePROMs/ePREMs can be difficult for patients with low language or computer literacy (B)	9	the PRO assessment was only available in English, which excluded patients who were non-English speakers. [30]
Completing ePROMs/ePREMs can be difficult for patients with physical or cognitive impairments (B)	8	In addition, patients' disease characteristics, cognitive, intellectual, and visual impairment also influenced patients' interest in using the ePSRM tool. “So, people that [have] developmental disabilities of course … they are not fully comprehending what the questions are trying to ask … so patients with dementia, patients with developmental disabilities …”. [28]
Patients not aware of purpose of ePROMs; needing to have rationale explained to them (B)	7	Patients cited multiple reasons as to why PROs had not been completed, with most citing a lack of understanding regarding their purpose. [41]
*Domain 3: Inner setting* Includes the features of the implementing organization that might influence implementation		
Regular training and education to build staff capacity and confidence with the ePROM system (F)	13	Many clinicians were not sufficiently familiar with Skindex-16 and its scoring and recognized that “training would help [to] understand it better.” Clinicians who were familiar with the tool were unsure how to use individual patient scores clinically. [43]
Integrating ePROMs/ePREMs into existing workflow routine or reconfiguring workflow to ensure integration of ePROMs (F)	14	Reconfigure workflow to ensure integration and access to PRO reports at clinical encounter. [36]
Staff or volunteers to facilitate ePROM collection (F)	10	Trained volunteers can assist patients with filling out the questionnaires before the doctor's visit. [33]
Buy-in of leadership (F)	9	Engaged leadership and a willing champion within each individual practice (e. g., quality improvement leader or office manager) helped to maintain momentum, to demonstrate the value of the data for improving quality of care, and to provide audit and feedback to providers and staff. [7]
Burden on staff facilitating ePROM collection (B)	8	The greatest burden was placed upon the registration staff, having to explain the purpose of the survey instruments and the importance of collection at each (and every) office visit. [9]
Time consuming and too many ePROMs/ePREMs (B)	8	Reasons why clinicians were not satisfied with the PROMs were too many PROMs. [35]
ePROMs increase time efficiency for clinicians in interview process and documentation (F)	8	Saving time and efficiency was also commonly mentioned. The implementation of the ePSRM helped reduce clerical time for both clinicians and nurses. [28]
*Domain 4: Characteristics of individuals* Characteristics of individuals involved in implementation that might influence implementation		
Improved prioritisation and targeting of patient-clinician communication (F)	13	Using PROs enabled psychologists to “know patient concerns upfront” and have “more targeted conversations with patients”. [30]
Not sure how ePROMs/ePREMs can inform clinical decisions (B)	10	The new users to CORE-Net only used the scores to inform them how to proceed in a limited way, for example, in terms of whether to continue work with the clients or to discharge them. They felt quite strongly that the ‘relationship with the client’ and their ‘own subjective measure’ based on their own experience is what informs and ‘influences’ the therapy process and their work, rather than the scores. [29]
Believing ePROMs/ePREMs not suitable/relevant/valuable (B)	9	Implementation was least successful when physicians did not find PROs valuable. [38]
Belief that ePROMs/ePREMs not clinically valid or lack accuracy (B)	5	Physicians were sceptical about the validity of PRO scores due to “little research around it [PRO] to help frame decision making or thoughts around it”. [30]
Belief that ePROMs/ePREMs duplicate clinical interview so are redundant (B)	5	Clinicians also expressed ambivalences in meso-level purposes of PROMs and PREMs use, asserting that their palliative expertise already encompassed routine conversational and observational quality of life assessments, and engendered robust interdisciplinary communication. [25]
Buy-in of clinical staff (F)	5	Individual interviews with nurses and physicians suggested that […] physician buy-in was key to successful PRO implementation. [38]
Clinician’s lack of knowledge and content of ePROMs/ePREMs (B)	5	Most Medical Assistants had little knowledge of the content of the assessment and thus had a hard time explaining how patients would reap direct benefits from completing it. “We [administer PROs] because we are required to do so”. [30]
*Domain 5: Process* Includes strategies or tactics that might influence implementation		
Presence of local staff champions to support/motivate peers and advocate for PROM usage (F)	9	A willing champion within each individual practice (e. g., quality improvement leader or office manager) helped to maintain momentum, to demonstrate the value of the data for improving quality of care, and to provide audit and feedback to providers and staff. [7]
Engagement/involvement of stakeholders (F)	9	For successful implementation, it takes effort to motivate all health care providers, administrative employees, and technology providers. [33]
Ongoing monitoring of implementation through regular audits, and regular feedback to users (F)	4	Audit and feedback: Performance reports designed with stakeholders and feedback to disease site teams for population-based QI; Systems to track progress and identify targets for improvement. [36]
Pre-implementation testing, especially of usability (F)	3	We found conducting a pilot phase to be very helpful in reducing any 'teething problems'. [33]
Project managers/coordinators skilled in knowledge translation and facilitating practice change (F)	3	Site coordinators skilled in knowledge translation and facilitating practice change were considered key to successful implementation. [36]

### CFIR domain: intervention characteristics

Within the included studies, stakeholders noted several areas in which ePROMs and ePREMs offered a relative advantage over alternative methods for gathering patient data. These advantages include *facilitating information extraction that might be overlooked or not uncovered in the consultation* [8, 24–27, 29, 34, 35, 38, 43], the potential for *earlier detection of issues* [24, 25, 29, 33, 35], how readily ePROMs/ePREMs *allow for monitoring changes* [24, 25, 28–36, 43], and the *ease with which comparisons can be made between patients and peers* [33]. Further advantages of the implementation of ePROMs are the *efficiency it allows in allocating finite hospital resources* [26], and the *reduced need for in-person consults* [26].

Regarding the complexity of the intervention, barriers emerged when *ePROMs were perceived by clinical staff as a time burden* due to *being delivered too often or being too lengthy or repetitive* [8, 24, 27, 30, 33–36, 39, 43]. Additionally, several studies have identified ePROMs can *flag too many concerns to be discussed within the limited clinic time available* [24, 25, 28, 30, 34, 38, 43]. Despite this, several studies acknowledged these complexities could be minimised by selecting an ePROM/ePREM platform with *user-friendly software and technology* that automatically captures, summarises and displays patient data [4, 9, 28–31, 35, 38].

Design quality and packaging of ePROMs were prominent constructs identified in 17 studies. Several studies have noted the benefits of ePROMs in *providing an insightful, easy to read patient summary or snapshot* [9, 29, 32, 35, 36, 43], *along with graphical visualisations to easily see trends* [8, 28–30, 32, 35, 38, 39, 41, 43]. It was also considered important to ensure *patients received ePROM results promptly after appointments* [26]. On the other hand, a significant barrier emerged when it was *difficult to visualise data or obtain a summary due to limited visualisation options* [30, 34]. The *inability to distinguish potentially abnormal results due to a lack of clearly defined threshold scores* was considered a barrier [9, 24, 29, 32, 36, 43]. In contrast, ePROM systems which *clearly identified results that exceeded threshold scores* and *supplied real-time notifications of those requiring urgent responses* was considered to support implementation success [30, 35].

Facilitators of implementation also included *adaptive technology to automatically trigger additional ePROMs, based upon earlier scores* [30, 33, 35, 36], and systems that *presented questions one at a time to patients, which was considered less overwhelming to patients than large numbers of paper forms* [28, 35]. The *automatic integration of ePROM results into electronic clinical notes* [30, 38], as well as *providing clinical staff with real-time access to ePROM completion status and results both prior to and during consults* were highlighted as facilitators [30, 35]. Other facilitators included the *provision of automatic reminders for clinicians to discuss ePROM results in consult time* [28, 34], and having *ePROMs available in different languages* [28, 30, 35, 36]. In contrast, *unreliable or unstable software and hardware* was identified as a barrier [4, 8, 9, 29–31, 33, 35, 40, 41]. Additional barriers to implementation included *ePROMs not being completed at a clinically meaningful timepoint* [30, 31, 33–35, 41], and the *prohibitive costs associated with implementing ePROMs/ePREMs* [7, 25, 33, 37, 39].

### CFIR domain: outer setting

Studies have provided mixed views on patient needs regarding the implementation of ePROMs and ePREMs. Some studies have acknowledged *patient frustration due to the lack of feedback from clinicians regarding ePROM results* [26, 28, 35, 43]. The *completion of ePROMs/ePREMs are considered difficult for patients with low language or computer literacy* [24–28, 30, 31, 40, 41], and for *patients with physical or cognitive impairments* [24–28, 30, 35, 40]. Alternatively, *ePROMs allowed patients to better communicate and prioritise their concerns with clinic visits* [8, 24, 26, 28, 30, 31, 33–36, 38, 41], while *amplifying the patients’ voice, thereby enhancing patient-centred care and shared decision-making* [24–26, 28, 29, 31–36, 39, 43]. Furthermore, increased *patient motivation to complete ePROMs was noted when patients felt they were contributing to ongoing research* [34], while also leading to *improved patient satisfaction and experiences* [4, 28, 37].

Some studies have identified implementation barriers around *patients’ level of comfort with, and access to digital technologies* [26, 28, 30, 35]. *Patients’ lack of awareness with regards to why they were being asked to complete the ePROMs* was considered another barrier [24, 27, 30, 33, 34, 36, 40], while several studies noted *educational resources for patients* (e.g. brochures, videos, and staff scripts) were necessary to facilitate completion [9, 34, 36, 41].

Ensuring *leadership buy-in* [4, 7, 8, 27, 28, 36–38, 41], and *alignment to the organisation’s strategic goals* [7, 8, 36], were both noted to assist in enabling effective ePROM/ePREM implementation. In contrast, stakeholders raised concerns about *organisational policies being either lacking entirely or conflicting with ePROM implementation goals* [7, 8, 36]. For example, a lack of congruency between organisational policies and implementation goals was identified in Schepers, et al. [7], with a lack of “formal ratification by management” to align policy and implementation work plans. When effectively implemented, ePROMs/ePREMs were noted to *allow for comparative analysis within or between systems and organisations* [4, 25, 32, 37], and the *ePROM/ePREM data could be used to justify continued or expanded funding towards health services* [25].

### CFIR domain: inner setting

The compatibility of ePROM/ePREMs with the existing workflow processes is mixed. Many studies have noted the benefits of *organisational workflow and time management* [24, 26–28, 34, 35, 39, 42], particularly when *ePROMs/ePROMs integrated seamlessly into existing workflow processes* [4, 7, 9, 27–31, 33, 35–37, 41, 43], and when *clinicians are able to access data in real time* [4, 28, 30, 32, 33, 36, 39, 43]. A small number of studies have also noted the benefits of providing *tablet computers to collect ePROMs at first contact with the patient (usually in the waiting room)* [9, 36, 40, 41]. Factors that challenge compatibility primarily relate to the *additional workload burden placed on staff* [7, 9, 30, 31, 35, 36, 39, 41]. More specifically, concerns were raised around the logistics of ePROMs *collection with respect to time and equipment limitations* [35, 38, 41, 43], *too many ePROMs/ePREMs to collect* [25, 30, 31, 33, 35, 38, 39, 41], and the *coordination of handing out, retrieving, and cleaning tablet devices* [9, 40]. Additional barriers highlighted were with respect to *staff turnover* and the *requirement of training new staff in use of ePROMs* [7, 8], where suggested counter strategies included direct *access to technical support staff* [28, 36], *regular staff training and education to build capacity and confidence* [8, 9, 24, 27–30, 33, 35–37, 39, 43], and *staff or volunteers to facilitate ePROMs collection* [7, 28–31, 33, 34, 36, 37, 39].

Tension to change was identified by clinician stakeholders as an issue *leading to anxiety and resistance to change* as barriers to ePROM/ePREM implementation [25, 29, 36, 43]. A *lack of staff incentives* was considered to aid stakeholder resistance [7, 30], however, ePROMs which were easily *integrated into existing electronic health records were thought to increase stakeholder readiness and potential for implementation success* [7, 8, 32, 33, 35, 36, 38].

Stakeholders viewed *leadership buy-in* as integral to ensuring a favourable implementation of ePROMs/ePREMs in the clinical setting [4, 7, 8, 27, 28, 36–38, 41]. Further facilitators also included the *ability to easily share ePROMs/ePREMs results with other clinicians* [25], along with the *presence of peers who are more familiar with ePROMs, enabling peer-to-peer learning* [28, 29, 31, 34, 36, 39]. This was considered to *encourage a cultural shift for clinicians to place similar value on ePROMs as other clinical data* [41]. Secondary uses of ePROMs/ePREMs data, such as *aiding clinical performance metrics* [4, 25, 36], and *supporting clinician self-reflection and peer supervision* [4, 29], have also facilitated implementation.

### CFIR domain: characteristics of individuals

There have been mixed findings regarding individuals’ attitudes towards and values placed on ePROMs and ePREMs. Many studies have identified the knowledge and belief systems of individual clinicians as implementation barriers, including *lack of knowledge of ePROM content* [7, 8, 30, 39, 43], *uncertainty in how ePROMs/ePREMs can inform clinical decisions* [7, 9, 25, 29, 30, 32, 33, 35, 41, 43], and *feelings of being overwhelmed by the excessive volume of reported ePREM data* [4]. Additionally, clinicians *beliefs that ePROMs/ePREMs are not clinically valid or lack accuracy* [24, 25, 28, 30, 38], and are *not suitable or relevant or valuable* [7, 8, 25, 30, 34, 35, 38, 39, 43], *duplicate the clinical interview* [8, 25, 30, 38, 43] or *fall outside the clinical scope of practice* [25, 43], were further implementation barriers. Nonetheless, stakeholders noted utilising ePROM results in clinic visits could *validate clinical perceptions about patient outcomes* [29], increase the *sense of objectivity that clinicians have when communicating with patients* [24, 33], and *improve prioritisation and targeting of patient–clinician communication* [44]. The *buy-in of clinical staff* was also noted by several studies to influence implementation outcomes [9, 31, 33, 37, 38].

### CFIR domain: process

Several studies have noted the benefit of having *project managers or coordinators who were skilled in both knowledge translation and facilitating practice changes* [8, 35, 36], coupled with the importance of *pre-implementation planning and testing of ePROM usability* [28, 33, 36]. In turn, the *engagement of multiple stakeholders* throughout the planning and implementation phases was considered a key to success [8, 27–29, 32, 33, 36–38]. Furthermore, the presence of *local staff champions to support/motivate peers and advocate for ePROMs/ePREMs usage* was an important facilitator [4, 8, 28, 30, 31, 33, 34, 36]. The use of *standardised workflow processes*, in conjunction with *regular audit and feedback*, enabled the identification and refinement of such processes in early implementation phases, supporting the scalability of ePROMs to additional health facilities [7, 8, 28, 36].

## Discussion

This systematic review synthesised literature pertaining to the facilitators and barriers that influence the effective implementation of electronic patient-reported outcomes and experience measures within existing clinical health settings. As identified in previous systematic reviews investigating ePROM implementation [3, 14], the facilitators and barriers highlighted in this review need to be contextualised within the local setting where the ePROMs/ePREMs are to be implemented. However, mapping the results to a theoretical framework such as CFIR, a common language and structure is established for organising the findings, which enables a more comprehensive and structured understanding of the factors that influence the success of an implementation.

This review highlighted that the point-of-care utilisation of ePROMs/ePREMs could strengthen the patient’s voice through improved communication and a focus on shared decision-making in the patient’s healthcare journey. Furthermore, this review builds on two previous systematic reviews, one of which examined the implementation of PROMs (not ePROMs specifically) in a variety of health settings and mapped facilitators and barriers to the CFIR framework [14], whereas, the other reviewed the benefits and disadvantages of ePROMs, without a specific focus on implementation [3]. In contrast, the current review focused specifically on understanding the factors that influence the implementation of ePROMs/ePREMs in clinical healthcare settings.

The data on ePREMs, though limited to just two studies [4, 25], suggests a great deal of overlap between the barriers and facilitators to implementing ePROMs and ePREMs. Specifically, 27 of 30 implementation factors identified within the two included studies investigating ePREMs were also found to be identified in the other studies investigating ePROMs. This degree of overlap exists despite ePREMs having quite different aims to ePROMs, measuring experiences rather than health outcomes. Nonetheless, the paucity of data highlights that ePREMs are a relatively under-utilised measure in the health landscape [4] and underscores the need for further research in this area.

When comparing the frequently mentioned code categories across the five CFIR domains found in this review with previous reviews a moderate number of categories were previously reported [3, 14, 44]. Previously identified facilitators included ePROMs/ePREMs amplifying patients’ voices, facilitating patient-centred care, and shared decision-making [14]; ePROMs allowing patients to better communicate and prioritise in clinic visits [14]; engagement/involvement of stakeholders; pre-implementation testing, especially usability [14]; and project managers/coordinators skilled in knowledge translation and facilitating practice change [14]. Barriers that were already identified in previous reviews included ePROMs as a time burden [14], lack of reliable and robust software and hardware [14], difficulty in completing ePROMs/ePREMs for patients with low language and computer literacy [3, 14, 44], burden on staff facilitating ePROM collection [14], time consumption and too many ePROMs/ePREMs [14, 44], not sure how ePROMs/ePREMs can inform clinical decisions [14], believing ePROMs/ePREMs not suitable/relevant/valuable [44], belief that ePROMs/ePREMs are not clinically valid or lack accuracy [14, 44], and clinicians’ lack of knowledge and content of ePROMs/ePREMs [14, 44]. Several bidirectional categories between previous reviews and the current review included: patients not aware of the purpose of ePROMs; need to have rationale explained to them [14, 44]; regular training and education to build staff capacity and confidence with the ePROM system [14, 44]; integrating ePROMs/ePREMs into existing workflow routine or reconfiguring workflow to ensure integration of ePROMs [14, 44]; improved prioritisation and targeting of patient–clinician communication [3, 14]; and the presence of local staff champions to support/motivate peers and advocate for PROM usage [14, 44].

When looking further afield within the existing literature, a major barrier remains in the integration and workflow of ePROMs/ePREMs into existing (or planned) electronic medical records which can significantly impact the effectiveness of implementation [45, 46]. Additionally, Gensheimer et al. [45], highlighted the importance of integration driven by health system leadership and supported by IT specialists. Briggs et al. identified a barrier arising from a lack of infrastructure, further reinforced by the lack of space to administer ePROMs/ePREMs, user-friendly electronic medical records for ePROM/ePREM integration, and equipment and resources [44].

This study provides unique insights into the implementation of ePROM/ePREMs, beyond those found in previous reviews. Approximately one-third of the most commonly occurring (top five) code categories in each domain of CFIR in the current study were not found in prior reviews, likely as a result of this review being inclusive of more recent literature and specifically focused on ePROMs and ePREMs. The new code categories were primarily mapped to the first two CFIR domains (intervention characteristics and outer setting) because ePROMs/ePREMs differ primarily in a key intervention characteristic—being electronic—and this has implications for how patients (outer setting) interact with them. With the growing application of technology in healthcare and, more directly, the adoption of electronic medical health records, these factors are now more evident in the reviewed literature.

Collectively, these findings highlight and contextualise facilitators and barriers to guide the implementation of ePROMs/ePREMs from the perspective of clinical practice. Recommendations for the implementation of ePROMs/ePREMs were provided as an implementation checklist (Additional File 4: Table S2). The use of such a checklist may ensure implementation efforts are targeted at ensuring the acceptance and sustainability of practice. This, in turn, supports the continued growth of embedding ePROM/ePREM within mainstream healthcare service provision through contextualised implementation across many Australian health sectors [47–49]. The use of the constructed implementation checklist from this review’s findings is thought to allow for greater utilisation of ePROMs/ePREMs in local health settings to better enhance patient-centred care and shared decision-making.

This systematic review has both strengths and limitations. First, most studies (n = 15) were conducted in three countries: the USA, Canada, and the Netherlands, whereas half (n = 12) of the studies implemented ePROMs/ePROMs in the clinical areas of either oncology [8, 33, 34, 36–39, 42] or orthopaedics [9, 30, 41]. As health care provision and funding vary vastly across the world, generalisability of findings to healthcare settings within other countries and other clinical cohorts may be limited. Further research across various countries, funding environments, and clinical populations are required.

Second, only two of the included studies investigated ePREM implementation [4, 25], the findings of this review with respect to implementation of ePREMs must be interpreted with caution because these identified factors are based on far fewer studies than those identified as influencing implementation of ePROMs. It should also be noted that although MMAT quality scores are presented as overall scores, this has been discouraged by some authors [17]. Nonetheless, it is anticipated the inclusion of study characteristics will enable transparency in the interpretation of the ratings. A further strength of this review is that the two reviewers independently synthesised the data, including independent consensus coding. Additionally, the CFIR determinant framework was used as a conceptual map in which code categories could be organised to produce actionable findings, thus enabling a better understanding of where efforts need to be directed when attempting to implement ePROMs/ePREMs. Furthermore, the pragmatic selection and design of strategies to implement the developed checklist by health services and organisations may be assisted using other frameworks (e.g., Expert Recommendations for Implementing Change Matching Tool) [50].

## Conclusion

This review provides a contemporary overview of the facilitators and barriers that influence the successful implementation of ePROMs and ePREMs in various health care settings. It highlights several factors that should be considered in future organisational processes when implementing ePROMs/ePREMs. We anticipate the findings of this review will be informative for clinical practitioners, public health officials, and other developing mechanisms for enhancing patient-reported outcomes. Further investigation of the facilitators and barriers identified in this review should be applied to other clinical areas to examine the generalisability of the findings. The relationship between clinician resistance to change in implementing ePROMs/ePREMs and their impact on the subsequent implementation of patient care and satisfaction also requires further investigation.

## Supplementary Information

**Additional file 1.** Search strategy for databases.

**Additional file 2.** Methodological quality scores for all included studies using Mixed-Methods Appraisal Tool (MMAT).

**Additional file 3.** **Table S1.** Full list of identified code categories, mapped to Consolidated Framework of Implementation Research (CFIR) constructs.

**Additional file 4.** **Table S2.** Implementation checklist.

## Data Availability

Full dataset and statistical codes are available upon request. The manuscript’s guarantor (BG) affirms that the manuscript is an honest, accurate, and transparent account of the study being reported, that no important aspects of the study have been omitted, and that any discrepancies from the study as originally planned (and, if relevant, registered) have been explained.

## Abbreviations

PROMs: Patient-reported outcome measures
ePROMs: Electronic patient-reported outcome measures
PREMs: Patient-reported experience measures
ePREMs: Electronic patient-reported experience measures
CFIR: Consolidated framework of implementation research
MMAT: Mixed-methods appraisal tool
PRISMA: Preferred reporting items for systematic reviews and meta-analyses

## References

[1] Teisberg E, Wallace S, O'Hara S (2020). Defining and implementing value-based health care: a strategic framework. Acad Med.

[2] Kingsley C, Patel S (2017). Patient-reported outcome measures and patient-reported experience measures. BJA Educ.

[3] Meirte J, Hellemans N, Anthonissen M, Denteneer L, Maertens K, Moortgat P (2020). Benefits and disadvantages of electronic patient-reported outcome measures: systematic review. JMIR Perioper Med.

[4] De Rosis S, Cerasuolo D, Nuti S (2020). Using patient-reported measures to drive change in healthcare: the experience of the digital, continuous and systematic PREMs observatory in Italy. BMC Health Serv Res.

[5] Watson L, Delure A, Qi S, Link C, Chmielewski L, Photitai É (2021). Utilizing patient reported outcome measures (PROMs) in ambulatory oncology in Alberta: digital reporting at the micro, meso and macro level. J Patient Rep Outcomes.

[6] Alo Health Welfare (2018). Australia's health 2018.

[7] Kwan BM, Sills MR, Graham D, Hamer MK, Fairclough DL, Hammermeister KE (2016). Stakeholder engagement in a patient-reported outcomes (PRO) measure implementation: a report from the SAFTINet practice-based research network (PBRN). J Am Board Fam Med.

[8] Schepers SA, Sint Nicolaas SM, Haverman L, Wensing M, Schouten van Meeteren AYN, Veening MA (2017). Real-world implementation of electronic patient-reported outcomes in outpatient pediatric cancer care. Psychooncology.

[9] Papuga MO, Dasilva C, McIntyre A, Mitten D, Kates S, Baumhauer JF (2018). Large-scale clinical implementation of PROMIS computer adaptive testing with direct incorporation into the electronic medical record. Health Syst (Basingstoke).

[10] Keith RE, Crosson JC, O’Malley AS, Cromp D, Taylor EF (2017). Using the consolidated framework for implementation research (CFIR) to produce actionable findings: a rapid-cycle evaluation approach to improving implementation. Implement Sci.

[11] Breimaier HE, Heckemann B, Halfens RJG, Lohrmann C (2015). The Consolidated framework for implementation research (CFIR): a useful theoretical framework for guiding and evaluating a guideline implementation process in a hospital-based nursing practice. BMC Nurs.

[12] Damschroder LJ, Aron DC, Keith RE, Kirsh SR, Alexander JA, Lowery JC (2009). Fostering implementation of health services research findings into practice: a consolidated framework for advancing implementation science. Implement Sci.

[13] Kirk MA, Kelley C, Yankey N, Birken SA, Abadie B, Damschroder L (2016). A systematic review of the use of the consolidated framework for implementation research. Implement Sci.

[14] Foster A, Croot L, Brazier J, Harris J, O'Cathain A (2018). The facilitators and barriers to implementing patient reported outcome measures in organisations delivering health related services: a systematic review of reviews. J Patient Rep Outcomes.

[15] Page MJ, McKenzie JE, Bossuyt PM, Boutron I, Hoffmann TC, Mulrow CD (2021). The PRISMA 2020 statement: an updated guideline for reporting systematic reviews. BMJ.

[16] Covidence systemtic review software. Melbourne, Australia: Veritas Health Innovation; [Available from: www.covidence.org

[17] Hong QN, Fàbregues S, Bartlett G, Boardman FK, Cargo M, Dagenais P (2018). The mixed methods appraisal tool (MMAT) version 2018 for information professionals and researchers. Educ Inf.

[18] Coates D, Coppleson D, Schmied V (2020). Integrated physical and mental healthcare: an overview of models and their evaluation findings. JBI Evid Implement.

[19] Gwet KL (2008). Computing inter-rater reliability and its variance in the presence of high agreement. Br J Math Stat Psychol.

[20] Gwet KL (2019) irrCAC: computing chance-corrected agreement coefficients (CAC). R package version 1.0. Available from: https://CRAN.R-project.org/package=irrCAC

[21] Team RC (2020) R: A language and environment for statistical computing. R Foundation for Statistical Computing Vienna, Austria. Available from: https://www.R-project.org/

[22] Krippendorff K (2019). Content analysis: an introduction to its methodology.

[23] Liberati A, Altman DG, Tetzlaff J, Mulrow C, Gøtzsche PC, Ioannidis JP (2009). The PRISMA statement for reporting systematic reviews and meta-analyses of studies that evaluate health care interventions: explanation and elaboration. J Clin Epidemiol.

[24] Fredericksen RJ, Tufano J, Ralston J, McReynolds J, Stewart M, Lober WB (2016). Provider perceptions of the value of same-day, electronic patient-reported measures for use in clinical HIV care. AIDS Care.

[25] Krawczyk M, Sawatzky R, Schick-Makaroff K, Stajduhar K, Öhlen J, Reimer-Kirkham S (2019). Micro–meso–macro practice tensions in using patient-reported outcome and experience measures in hospital palliative care. Qual Health Res.

[26] Nielsen AS, Appel CW, Larsen BF, Kayser L, Hanna L (2021). Patient perspectives on digital patient reported outcomes in routine care of inflammatory bowel disease. J Patient Rep Outcomes.

[27] Short D, Fredericksen RJ, Crane HM, Fitzsimmons E, Suri S, Bacon J (2022). Utility and impact of the implementation of same-day, self-administered electronic patient-reported outcomes assessments in routine HIV care in two North American clinics. AIDS Behav.

[28] Spaulding A, Nordan L, Blanchfield L, Asiedu GB, Saltivan J, Pecenka S (2019). Qualitative study of implementation of patient self-reported measures in a consultation-liaison psychiatry practice. J Eval Clin Pract.

[29] Unsworth G, Cowie H, Green A (2012). Therapists’ and clients’ perceptions of routine outcome measurement in the NHS: a qualitative study. Couns Psychother Res.

[30] Zhang R, Burgess ER, Reddy MC, Rothrock NE, Bhatt S, Rasmussen LV (2019). Provider perspectives on the integration of patient-reported outcomes in an electronic health record. JAMIA Open.

[31] Amini M, Oemrawsingh A, Verweij LM, Lingsma HF, Hazelzet JA, Eijkenaar F (2021). Facilitators and barriers for implementing patient-reported outcome measures in clinical care: an academic center's initial experience. Health Policy.

[32] Burton SV, Valenta AL, Starren J, Abraham J, Nelson T, Kochendorfer K (2022). Examining perspectives on the adoption and use of computer-based patient-reported outcomes among clinicians and health professionals: a Q methodology study. J Am Med Inform Assoc.

[33] Dronkers EAC, Baatenburg de Jong RJ, van der Poel EF, Sewnaik A, Offerman MPJ (2020). Keys to successful implementation of routine symptom monitoring in head and neck oncology with "Healthcare Monitor" and patients' perspectives of quality of care. Head Neck.

[34] Duman-Lubberding S, van Uden-Kraan CF, Jansen F, Witte BI, Eerenstein SEJ, van Weert S (2017). Durable usage of patient-reported outcome measures in clinical practice to monitor health-related quality of life in head and neck cancer patients. Support Care Cancer.

[35] Teela L, van Muilekom MM, Kooij LH, Gathier AW, van Goudoever JB, Grootenhuis MA (2021). Clinicians' perspective on the implemented KLIK PROM portal in clinical practice. Qual Life Res.

[36] Howell D, Rosberger Z, Mayer C, Faria R, Hamel M, Snider A (2020). Personalized symptom management: a quality improvement collaborative for implementation of patient reported outcomes (PROs) in 'real-world' oncology multisite practices. J Patient Rep Outcomes.

[37] Li M, Macedo A, Crawford S, Bagha S, Leung YW, Zimmermann C (2016). Easier said than done: keys to successful implementation of the distress assessment and response tool (DART) program. J Oncol Pract.

[38] Rotenstein LS, Agarwal A, O'Neil K, Kelly A, Keaty M, Whitehouse C (2017). Implementing patient-reported outcome surveys as part of routine care: lessons from an academic radiation oncology department. J Am Med Inform Assoc.

[39] Trautmann F, Hentschel L, Hornemann B, Rentsch A, Baumann M, Ehninger G (2016). Electronic real-time assessment of patient-reported outcomes in routine care-first findings and experiences from the implementation in a comprehensive cancer center. Support Care Cancer.

[40] Hanmer J, Ray KN, McCracken P, Ferrante L, Wardlaw S, Fleischman L (2021). Uptake of an integrated electronic questionnaire system in community pediatric clinics. Appl Clin Inform.

[41] Bhatt S, Davis K, Manning DW, Barnard C, Peabody TD, Rothrock NE (2020). Integration of patient-reported outcomes in a total joint arthroplasty program at a high-volume academic medical center. J Am Acad Orthop Surg Glob Res Rev.

[42] Bärlund M, Takala L, Tianen L, Kellokumpu-Lehtinen PL (2022). Real-world evidence of implementing eHealth enables fluent symptom-based follow-up of a growing number of patients with breast cancer with the same healthcare resources. Clin Breast Cancer.

[43] Taliercio VL, Snyder AM, Biggs AM, Kean J, Hess R, Duffin KC (2022). Clinicians' perspectives on the integration of electronic patient-reported outcomes into dermatology clinics: a qualitative study. Qual Life Res.

[44] Briggs MS, Rethman KK, Crookes J, Cheek F, Pottkotter K, McGrath S (2020). Implementing patient-reported outcome measures in outpatient rehabilitation settings: a systematic review of facilitators and barriers using the consolidated framework for implementation research. Arch Phys Med Rehabil.

[45] Gensheimer SG, Wu AW, Snyder CF (2018). Oh, the places we'll go: patient-reported outcomes and electronic health records. Patient.

[46] Stover AM, Haverman L, van Oers HA, Greenhalgh J, Potter CM (2021). Using an implementation science approach to implement and evaluate patient-reported outcome measures (PROM) initiatives in routine care settings. Qual Life Res.

[47] Morris ME, Brusco N, Woods J, Myles PS, Hodge A, Jones C (2021). Protocol for implementation of the 'AusPROM' recommendations for elective surgery patients: a mixed-methods cohort study. BMJ Open.

[48] Girgis A, Bamgboje-Ayodele A, Rincones O, Vinod SK, Avery S, Descallar J (2022). Stepping into the real world: a mixed-methods evaluation of the implementation of electronic patient reported outcomes in routine lung cancer care. J Patient Rep Outcomes.

[49] Thompson C, Sansoni J, Morris D, Capell J, Williams K (2016) Patient-reported outcome measures: an environmental scan of the Australian healthcare sector

[50] Perry CK, Damschroder LJ, Hemler JR, Woodson TT, Ono SS, Cohen DJ (2019). Specifying and comparing implementation strategies across seven large implementation interventions: a practical application of theory. Implement Sci.

