# Supplementary material for: Facilitators and barriers to implementing electronic patient-reported outcome and experience measures in a health care setting: a systematic review
Source: J Patient Rep Outcomes. 2023 Feb 14;7:13. doi: 10.1186/s41687-023-00554-2 (PMC9928985; doi:10.1186/s41687-023-00554-2)
Supplement: Supplementary file 1 — Additional file 1. Search strategy for databases. [file 41687_2023_554_MOESM1_ESM.docx]

Additional File 1. The search strategy.

| **Search** | **Query** |
| --- | --- |
| **#1** | PROM [Text Word] OR PROMS [Text Word] OR eprom [Text Word] OR eproms [Text Word] OR epro [Text Word] OR patient reported outcome*[Text Word] OR "Patient Reported Outcome Measures"[Mesh] |
| **#2** | prem [Text Word] OR prems [Text Word] OR epre [Text Word] OR eprem [Text Word] OR eprems [Text Word] OR patient reported experience measure*[Text Word] |
| **#3** | #1 OR #2 |
| **#4** | Implement*[Text Word] |
| **#5** | #3 AND #4 |
| **#6** | Electronic [Text Word] OR digital [Text Word] OR mHealth [Text Word] |
| **#7** | #5 AND #6 |
