# Supplementary material for: Facilitators and barriers to implementing electronic patient-reported outcome and experience measures in a health care setting: a systematic review
Source: J Patient Rep Outcomes. 2023 Feb 14;7:13. doi: 10.1186/s41687-023-00554-2 (PMC9928985; doi:10.1186/s41687-023-00554-2)
Supplement: Supplementary file 4 — Additional file 4. Table S2. Implementation checklist. [file 41687_2023_554_MOESM4_ESM.docx]

**Additional file 4: ePROM and ePREM Implementation checklist**

**N.B. Checklist items mapped to Consolidated Framework of Implementation Research domains**

| **ePROM and ePREM Implementation checklist** |
| --- |
| **Pre-implementation** |
| **Intervention characteristics**  **(Relative advantage stakeholders’ perception of the advantage of implementing the innovation versus an alternative solution, complexity, design quality, packaging & cost)** |
| - Ensure user-friendly software, with stable and reliable hardware - Provide a very clear visualisation and summary of ePROM data in Electronic Health Records - Ensure ePROMs are available in different languages - Decrease the time burden of ePROMs by ensuring they are not too long or repetitive - Ensure a simplified and timely process for the distribution and recovery of patient passwords - Present ePROM questions one at a time to patients, to appear less overwhelming - Have clearly defined threshold scores to help clinicians differentiate between normal and abnormal ePROM results - Streamline the number of concerns identified by ePROMs to ensure timely response - Trigger additional ePROMs automatically were indicated based on ePROM responses - Notify clinicians automatically and in real-time of ePROM results that require an urgent response - Secure sufficient financial resources to invest in implementation (including software, hardware, and personnel) |
| **Outer setting**  **(Patient needs, external policies & incentives)** |
| - Ensure support strategies are in place to assist patients with physical or cognitive impairments or low language/computer literacy - Provide alternatives for patients who do not have access to the internet or provide a paper option for patients who prefer it - Allow ePROM data to be used for the purpose of comparative analysis between systems and organisations |
| **Inner setting**  **(Networks & communications, culture, implementation climate, tension for change, compatibility, organisational incentives & rewards, goals and feedback, learning climate, readiness for implementation, leadership engagement, available resources, access to knowledge & information)** |
| - Automatically integrate results of ePROMs into electronic clinical notes - Create and distribute educational resources for patients on the purpose of ePROMs/ePREMs and instructions for use (e.g., brochures, videos) - Accommodate discussion of ePROM results within clinic time - Present ePROMs to patients at appropriate times (at home or in the waiting room), giving sufficient time to ensure completion prior to consult - Ensure clinicians have real-time access to their patients’ ePROM results (and competition status) prior to consult - Consider how to integrate ePROMs/ePREMs into existing workflow routine, or how to reconfigure workflow to ensure integration of ePROMs/ePREMs (e.g., reviewing ePROM data prior to consult) - Provide regular opportunities for staff training and education to build capacity and confidence with the ePROM system |
| **Process**  **(Planning, implementation leaders, executing, reflecting & evaluating)** |
| - Ensure early engagement/involvement of stakeholders - Employ project managers/coordinators who are skilled in knowledge translation and facilitating practice change |
| **Implementation** |
| **Intervention characteristics**  **(Relative advantage stakeholders’ perception of the advantage of implementing the innovation versus an alternative solution, complexity, design quality, packaging & cost)** |
| - Provide reminders for clinicians to discuss ePROM results in clinic time - Provide tablet computers to patients at the first point of contact (e.g., waiting room) to facilitate ePROM collection - Ensure completion of ePROM/ePREMs in a timely fashion (prior to consult at home or in the waiting room) - Ensure the presence of existing electronic health records into which ePROMs can easily be integrated |
| **Outer setting**  **(Patient needs, external policies & incentives)** |
| - Ensure patients receive timely feedback on ePROM results and can review the summary of their data after appointments - Implement strategies to support patients who experience anxiety undertaking ePROMs (e.g., due to listing expected symptoms) |
| **Inner setting**  **(Networks & communications, culture, implementation climate, tension for change, compatibility, organisational incentives & rewards, goals and feedback, learning climate, readiness for implementation, leadership engagement, available resources, access to knowledge & information)** |
| - Consider the logistics of, and provide an efficient process for, handing out, retrieving, and cleaning tablet computers - Allow ePROMs to be used as clinician performance metrics, and be analysed at all levels of an organization - Allow ePROM/ePREM data (scores and collection rates) to be utilised by clinicians to self-reflect and as a supervision tool - Ensure the availability of technical support staff - Consider the use of staff or volunteers to facilitate ePROM collection |
| **Process**  **(Planning, implementation leaders, executing, reflecting & evaluating)** |
| - Ensure capability to rapidly iterate on stakeholder feedback, facilitated through the flexible implementation |
| **Sustainability** |
| **Intervention characteristics**  **(Relative advantage stakeholders’ perception of the advantage of implementing the innovation versus an alternative solution, complexity, design quality, packaging & cost)** |
| - Ensure ePROM system provides clinicians with a snapshot or summary of patient data that is insightful and easy to read - Use ePROM data to facilitate comparisons between patients and peers |
| **Outer setting**  **(Patient needs, external policies & incentives)** |
| - Ensure the organisation’s goals align with implementation goals - Allow for the use of ePROM/ePREM data to justify continued or expanded funding toward health services - Ensure implementation goals and policies are not conflicting |
| **Inner setting**  **(Networks & communications, culture, implementation climate, tension for change, compatibility, organisational incentives & rewards, goals and feedback, learning climate, readiness for implementation, leadership engagement, available resources, access to knowledge & information)** |
| - Facilitate the sharing of ePROM/ePREM results between clinicians - Ensure the necessary workplace and organisational adjustments are in place - Have a clear division of responsibility for who reviews ePROMs, and when - Encourage a cultural shift to value ePROM/ePREM data as much as other patient data - Consider the effect of staff turnover and ensure adequate replacement of staff when they leave - Provide adequate support, training, and resources for new staff - Provide incentives to encourage staff to use ePROMs - Consider the benefit of the presence of peers who are more familiar with ePROMs, enabling peer-to-peer learning |
| **Characteristics of individuals**  **(Knowledge & beliefs about the intervention & individual Identification with organisation)** |
| - Encourage buy in of clinical staff - Ensure there is no fear of staff being judged on ePROM results by peers |
| **Process**  **(Planning, implementation leaders, executing, reflecting & evaluating)** |
| - Ensure patients receive results of ePROM/ePREMs promptly after the appointment - Consider collaboration in implementation between sites, utilising a program coordinator - Have a standardised implementation process in place, with specific action items revised as new issues are identified, to streamline implementation for future sites - Continually monitor implementation through regular audits, and provide regular feedback to users |
